# Smart Wearables in the Lens of Aging: Results From the ROAMM Study

**DOI:** 10.1093/geroni/igaa057.2894

**Published:** 2020-12-16

**Authors:** Mamoun Mardini, Todd Manini, Jennifer Schrack

## Abstract

Continuous, long-term monitoring with remote capabilities using wearable technology is ideal for capturing information about patient/participant symptoms synced to sensor-based information. The Real-time Online Assessment and Mobility Monitor (ROAMM) is a smartwatch framework configured to collect data in free-living settings from both sensor-based (location and movement) and responses to symptom notifications through a visual display. The symposium presents the overall framework and preliminary findings from a demonstration study in older adults with knee osteoarthritis. Karnati will present the general framework of ROAMM explaining the data flow from the smartwatch to end users (clinicians and research). He will highlight components in the design that makes the framework unique and highly flexible to serve different studies with different research questions. Rouzaud evaluated satisfaction, usability and compliance wearing a smartwatch and using the ROAMM app. Participants were compliant to ecological prompts about pain, fatigue and mood three times a day (82.5% compliance rate). Additionally, > 70% reported being satisfied with the function/usability and comfort with using ROAMM and wearing the smartwatch. Mardini examined the temporal relationship between ecological pain and derived life-space mobility features from Global Positioning System coordinates. Results suggested that higher level of knee pain in older adults was associated with lower life-space mobility. Manini examined physician perception towards an electronic health record (EHR) graphical interface of top ranked patient attributes of pain, falls, hydration and mobility patterns. Results indicated a relatively high level of usability of the EHR interface depicting smartwatch data.

